# On Emetics in Diabetes Mellitus

**Published:** 1814-12

**Authors:** J. R. Walker

**Affiliations:** Huddersfield


### *For the Medical and Physical Journal.*

### *On Emetics in Diabetes Mellitus;* by Dr. J. R. WALKER, of Huddersfield.

THE case here detailed is intended to show the successful exhibition of Emetics, repeated every other morning, in Diabetes Mellitus, in a patient who had been sinking for many months, without relief from the ordinary remedies. The patient, Thomas Ainsley, æt. 21, a resident in this neighbourhood, had laboured for two years past with symptoms of dyspepsia, but during the last year he had felt more particularly a craving and uneasiness of stomach, with polydipsia. The amount of the urine had varied at different times since April last, but thinks it has seldom been less than six or eight pints a-day. On the 4th of September last it exceeded eleven pints, was somewhat of a greenish tinge, and left a residuum like treacle on evaporation. It also fermented with yeast, and had very much of that hay-like odour alluded to by Dr. Latham in his Treatise on Diabetes. Without entering into any scruples whether the stomach was primarily or only sympathetically affected, I resolved to combat what appeared to me to be one essential part of the disease in its present stage at least, viz. the morbid affection of the stomach, not, however, with any flattering hope of success, the same treatment with slight variation having so frequently failed. The emetic powder, which I ordered, was that so much extolled by Dr. Senter in phthisis pulmonalis, viz. seven grains each of Sulphas Cupri and Ipecacuanha, to be taken every other morning fasting, without drinking any thing afterward. He commenced taking the first powder on the 4th of September, at which time his urine rather exceeded eleven pints. He was ordered also to take the following powder three times a-day.

R. Ferri.

℞. Ferri Ammoniati.

Uvæ Urs. Contus. āā ℈j. M. ft. Pulvis Ope Vehiculi crassi exhibendus.

He kept his bowels constantly open by means of the phosphate of soda, and was ordered to confine himself strictly to animal diet.

I saw him in about a fortnight after, at which time, though his urine far exceeded the liquid ingesta, it was diminished nearly two pints and a half. Excepting the addition of the dilute nitric acid, with which he acidulated his drink, and the addition of a few grains to his emetic powder, which had lost some of its strength by long use, he persevered in the same course. Before the expiration of a month, his urine was much less than eight pints; his voracity and thirst, with parched heat of the surface, were much on the decline. In six weeks from the first exhibition of the emetics, the urine fell to six pints. He kept no account either of his liquid or solid ingesta that could be relied upon, but it was evident that the functions of the digestive organs began to be more natural, and his spirits to revive. On the 5th of November his urine was diminished nearly to four pints, being more than seven pints less than it was in August, and very much improved in colour, smell, and other properties.

Here, therefore, however theorists may differ as to the primary seat of the disease, it was evidently first arrested by a class of medicines which we have been accustomed to consider as possessing at least a primary agency on the stomach, for I lay but small stress on the efficacy of his other medicines, most of which he had made trial of before I became concerned for him. I am fully aware that instances are not wanting of similar success, where the disease disappeared for a time, but returned in a few months, and the patients have died at last of it; but in so desperate a disease it is the duty of a practitioner to register every, even partial, success which he obtains, as affording the best prospect of ultimately attaining more efficacious and radical relief. The ingenuity of hypotheses has racked itself to obtain some plausible theory of this disease, one set attributing to a vitiation of the gastric juice or defective classification and assimilation, a second to a colliquation of the fluids, a third to a retrograde action of the urinary lymphatics, some to a derangement or relaxation of the kidneys, others to a defect of vital heat. One point, however, seems to be established by universal experience, that whatever has succeeded in restoring the functions of the stomach to their natural state, has never failed to alleviate some of the most obstinate and essential symptoms that constitute this formidable disorder.

*Huddersfield, Nov. 5th.* J. R. WALKER, M.D.

